# Knocking Out of CEACAM1 Can Reduce Oxidative Stress and Promote Cell Proliferation in the HPMVECs under Hypoxia

**DOI:** 10.1155/2022/1748793

**Published:** 2022-06-26

**Authors:** Zhixuan Li, Xiaokang He, Xueting Zhang, Junhua Zou, Hao Li, Jing Wang

**Affiliations:** ^1^Department of Cardiology, The First Affiliated Hospital of Kunming Medical University, Kunming, Yunnan, China; ^2^Department of Geriatric Cardiology, The First Affiliated Hospital of Kunming Medical University, Kunming, Yunnan, China

## Abstract

Pulmonary hypertension (PH) induced by hypoxia is common in clinical practice and often suggests a poor prognosis. The oxidative stress and proliferation of pulmonary vascular endothelial cells caused by hypoxia are the major mechanisms involved in the pathophysiology of PH. It has been reported in recent years that the carcinoembryonic antigen-related cell adhesion molecule 1 (CEACAM1) promotes angiogenesis. In this study, normal human pulmonary microvascular endothelial cells (HPMVECs) and HPMVECs with stable knockout of CEACAM1 by CRISPR-Cas9 were subjected to oxygen-glucose deprivation/reperfusion (OGD/R) to induce hypoxic conditions. JC-1, ROS, and cell cycle profile were analyzed for each cell line and controls, using flow cytometry. A tube formation assay was used to detect angiogenesis, along with expression levels of CEACAM1, TNF-*α*, VEGF, VEGFR-2, p-P38/P38, and CyclinD1 proteins (to distinguish profiles of angiogenic growth and cell proliferation). We observed increased expression of CEACAM1 in HPMVECs after OGD/R, while ROS production was reduced and mitochondrial membrane potential was increased after OGD/R in CEACAM1^−/−^ HPMVECs. Furthermore, we observed increased cell division in CEACAM^−/−^ HPMVECs, accompanied by enhanced angiogenesis and reduced TNF-*α* protein expression and increased VEGF, VEGFR-2, and CyclinD1 expression. Together, these data suggest that upregulation of CEACAM1 in HPMVECs under hypoxic conditions may damage cells by increasing oxidative stress and inhibiting cell proliferation.

## 1. Introduction

Pulmonary hypertension (PH) is a clinical and pathophysiological syndrome caused by a wide range of diseases/causes and by diverse pathogenic processes which result from structural or functional changes in the pulmonary vasculature and lead to increased pulmonary vascular resistance and pulmonary arterial pressure. Despite great advances in diagnosis and treatment, PH remains a chronic cardiopulmonary disease that severely threatens human health. Data from a large, multisite observational research registry in the US (US-REVEAL) suggested that the estimated 7-year survival of PH was only 49% [1]. PH is a highly heterogeneous syndrome, in which cases are classified into five major groups, based on criteria including etiology, mechanism of disease pathology, hemodynamics, and prognosis. The most common subtype observed in many clinical settings is PH caused by lung disease and/or hypoxia, due to long-term destruction of lung parenchyma or interstitium and subsequent damage to the pulmonary vascular bed. Its pathophysiology is complex and includes hypoxic pulmonary vasoconstriction/vascular remodeling, vascular endothelial, and smooth muscle dysfunction, inflammation, and hypercoagulability [2, 3]. The carcinoembryonic antigen-related cell adhesion molecule 1 (CEACAM1), a member of the immunoglobulin superfamily that regulates autoimmunity and antitumor immunity [4], has been implicated in PH disease pathology. Recently, CEACAM1 has also been reported to play a role in promoting angiogenesis [5]. The purpose of the current study was to determine how CEACAM1 may affect oxidative stress and cellular proliferation in human pulmonary microvascular endothelial cells (HPMVECs) under hypoxic conditions. To do this, we generated HPMVECs expressing only a mutated version of CEACAM1 using lentiviral packaging method and compared them to wild type HPMVECs to assess their replicative and differentiation potential, as well as markers of oxidative stress.

## 2. Methods

### 2.1. Screening for Suitable Conditions for HPMVEC Growth for OGD/R Assay

HPMVECs cells (ScienCell, USA) were grown in ECM complete media supplemented with 5% fetal bovine serum (Gibco, USA), 1% penicillin-streptomycin (Meilun Bio, China), and 1% endothelial cell growth factor (Proteintech, Wuhan Sanying) at 37°C with 5% CO_2_. Growth media was changed every 1-2 days. Cells were passaged when cell density reached >90%. Cells were collected after three to four passages and seeded in 96-well plates at a density of 2.5 − 3 × 10^4^ cells/well for experiments. Equivalent plates were separated into two groups of three replicates each: for study under control or OGD/R conditions. For induction of OGD/R, cell culture media was exchanged for glucose-free DMEM medium (Meilun Bio, China) containing 94% N2, 1% O2, and 5% CO2, for 2, 4, and 8 hours, respectively, and then restored to the same standard growth media used for control groups throughout the experiment, for 12 h of reoxygenation and reglycation. After treatment, cell viability was monitored by MTT assay, and morphological changes to cells were observed under 100 magnification, using an inverted microscope. Preliminary experiments showed that cell viability was reduced and apoptosis increased after 8 hours of OGD conditions. Therefore, these conditions of OGD for 8 h and recovery following OGD for 12 h were selected for use subsequent experiments.

### 2.2. Construction of CEACAM1^−/−^ Plasmid Vector

According to the literature, we chose to construct the vector of CEACAM1^−/−^ (Oligo sequences: AUCAAACACAGAUAGUGUGU) purchased from Changsha Aonuo Gene Company, China.

### 2.3. Construction of CEACAM1^−/−^ and Negative Control Stable Cell Lines

CEACAM1-/- and control lentiviruses were used to infect HPMVECs cells, and 18-24 hours before lentivirus transfection, adherent cells were plated into 6-well plates at 3 × 10^5^/well. Lentivirus infection was carried out after approximately one cell doubling time, on approximately 6 × 10^5^/well, when cell confluence had reached approximately 70%. Growth medium was replaced with 2 mL of fresh medium containing 8 *μ*g/mL polybrene, and an appropriate amount of virus suspension was added. Cells were incubated with lentiviral suspension for 24 hours at 37°C, and the virus-containing medium was replaced with fresh medium. Lentiviruses contain fluorescent proteins. Generally, obvious fluorescent expression can be seen 48 hours after transfection, and more obvious after 72 hours. Puromycin selection was used to select for infected cells under empirically defined conditions (MOA: 30 concentration, which results in >90% fluorescent cells in normal growth conditions).

### 2.4. Experimental Grouping

The HPMVECs were split into four groups: control group, OGD/R group, OGD/R + NC group, and OGD/R + CEACAM1^−/−^ group. The OGD/R group was cultured according to the preexperimental screening conditions; the control group was not treated with OGD/R, while CEACAM1^−/−^ and NC stable cell lines were treated with OGD/R.

### 2.5. Detection of Protein Expression of CEACAM1, TNF-*α*, VEGF, VEGFR-2, p-p38/p38, and CyclinD1 by Western Blot

At the conclusion of treatment, cells were washed once with 1× PBS and trypsinized and collected by centrifugation, and cell pellets were washed with PBS, before pelleting for protein extraction. Total protein was extracted using a total protein extraction kit (Servicebio, China, Wuhan). Protein concentrations were measured by BCA (Biyuntian, China) assay, and remaining pellets were denatured by boiling in SDS loading buffer for 12 min. Twenty micrograms of total protein from each lysates was loaded into a lane in a 10% polyacrylamide gel for SDS-PAGE and then transferred to a membrane at 250 mA for 7 h. Membranes were rinsed three times with 1× TBST for 5 minutes each and then blocked with 5% nonfat milk/1X TBST for 90 minutes before addition of fresh blocking solution containing specific primary antibody. Concentrations used for each antibody are as follows: (CaV1.2, Huan Biology, 1 : 1000, 249 kDa; Cx40, Huaan Biology, 1 : 1000, 40 kDa; SUR2A, Affinity, 1 : 1000, 174 kDa; Kir6.2, Huaan Biology, 1 : 1000, 44 kDa; *β*-actin, Abcam, 1 : 5000). Blots were incubated in primary antibody overnight at 4°C with shaking, then washed four times with 1× TBST. The membranes were then incubated for 2 hours at room temperature with horseradish peroxidase- (HRP-) conjugated secondary antibodies before being rinsed four times with 1× TBST for 10 minutes each. Use ECL chromogenic solution (Affinity Biosciences, Jiangsu, China), and then fluorescence was detected using chemiluminescence imaging system (Shanghai Qinxiang Scientific Instrument Co., Ltd.). Protein levels were quantified by fluorescent signal and normalized to *β*-actin antibody as an internal reference.

### 2.6. Cell Cycle Detection

For cell cycle analysis, following treatment, cells were washed with 1× PBS and harvested from culture wells using trypsin. Cells (1 × 10^6^/mL) were harvested and centrifuged and fixed in a solution of 75% precold ethanol for 30 minutes at 4°C, followed by centrifugation at 1,000 rmp for 3 to 5 minutes. After removing any residual ethanol, cells were washed and resuspended in 1× PBS, then 500 *μ*L prepared dye working solution (480 *μ*L staining buffer, 10 *μ*L of 50 *μ*g/mL PI staining solution, and 10 *μ*L RNase A stock solution at 50 *μ*g/mL) was added. The cells were incubated in staining solution in total darkness for 30–60 min at 37°C and tested on the flow cytometer.

### 2.7. Detection of Reactive Oxygen Species (ROS) in Cells

To detect reactive oxygen species (ROS) in experimental samples, we used an ROS detection kit (Biyuntian, China). Cells from all 4 experimental groups (control, OGD/R, OGD/R + NC, and OGD/R + CEACAM1^−/−^) were analyzed by flow cytometer (FACSCalibur, BD), performing ROS detection at an excitation wavelength of 488 nm and a 525 nm emission wavelength. Based on background staining level in negative control sample, DCF expression level was set and used to gate cells.

### 2.8. Assessment of Mitochondrial Membrane Potential Using JC-1 Staining

We used a JC-1 detection kit (Biyuntian, China) to count and plate the treated control group, OGD/R group, OGD/R + NC group, and OGD/R + CEACAM1^−/−^ group cells. To measure JC-1, 1x JC-1 staining buffer was diluted from stock using distilled water and placed in an ice bath. After incubation at 37°C, cell supernatant was aspirated and washed with ice-cold 1x JC-1 staining buffer for 2. Cell culture medium containing serum and phenol red was added after staining, and cells were analyzed by flow cytometry (FACSCalibur, BD), at 488 nm excitation wavelength and 525 nm emission wavelength.

### 2.9. Tube Formation Assay

For tube formation assays, Matrigel was precoated onto 48-well plates before treatment. After treatment appropriate to each experimental group, cells were washed once with 1× PBS and digested with 0.25% pancreatin. The resuspended cells were filtered using a 100 *μ*m cell strainer to remove large cell aggregates and counted, and cells were seeded onto 48-well plates, at a final density of 4 × 10^4^ cells/well. After a 3 h incubation at 37°C, cells were imaged at 100× magnification using light microscopy. The number and the total length of formed tubes, nodes, and crossovers were measured using ImageJ.

## 3. Results

### 3.1. Establishment of Experimental Conditions for OGD/R with HPMVECs

**Figure 1:**
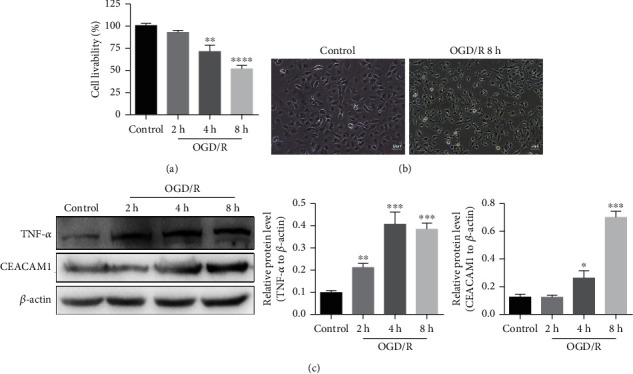
Culture of HPMVECs under OGD/R results in loss of cell viability and increased expression of CEACAM1 and TNFa. (a) Increased time under hypoxic conditions resulted in decreased cell viability in wild type HPMVECs. Assays were performed as biological replicates of three. The 8 h OGD-treated group exhibited significantly lower viability than 4 h OGD-treated group (*p* < 0.05). (b) Extended hypoxia yields smaller and more spherical cells and lower cell numbers compared to controls. (c) Increased time under hypoxic conditions leads to increased expression CEACAM1 and TNF-*α* protein.

We used the CCK-8 kit (Japan Colleagues Research Institute) assay to investigate the metabolic activity of our cells. To establish conditions for this assay, we assessed the cell viability of HPMVECs maintained under OGD for 2 h, 4 h, and 8 h, followed by reoxygenation for 12 h. As shown in Figure 1(a), relative to the viability of the control group (set to 100%), cells maintained under OGD for 2, 4, and 8 hours had relative viability scores of 100.000% ± 1.651%, 70.690% ± 4.384%, and 51.400 ± 2.558%, respectively. The viability of both 4 h and 8 h OGD-treated groups was diminished, with the latter decreased significantly (*p* < 0.05; Figure 1(a)). We observed that OGD treatment also caused the cells to become smaller and more spherical, accompanying the reduction in cell number as time under hypoxic conditions increased (Figure 1(b)). Interestingly, we found that extension of OGD treatment from 2 to 4 and 8 h resulted in concomitant increases in expression levels of CEACAM1 and TNF-*α* proteins (Figure 1(c)).

### 3.2. Construction and Validation of CEACAM1^−/−^ and Infection-Control HPMVEC Cell Lines

**Figure 2:**
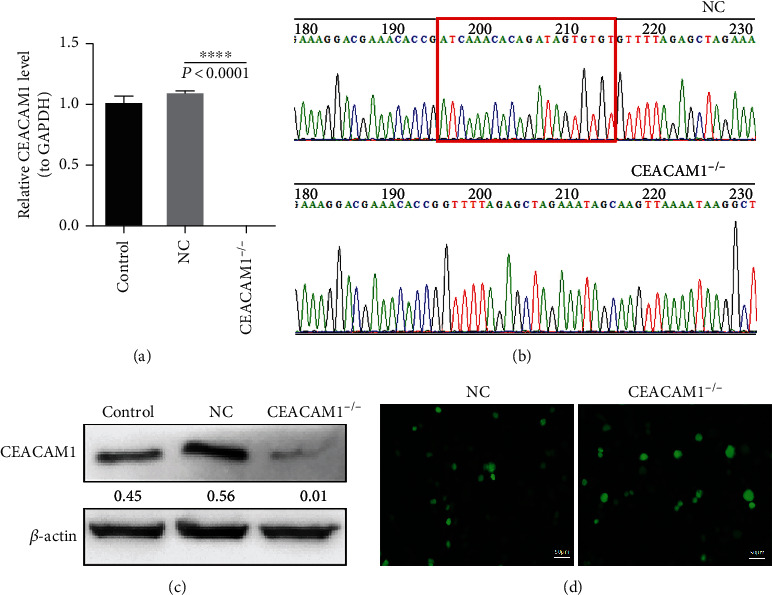
HPMVECs infected with mutated CEACAM construct exhibit reduced CEACAM protein expression. (a) Quantification of CEACAM1 expression in control and mutant HPVMECs. (b) CEACAM1 knockout was confirmed by DNA sequencing. The mutation is highlighted by a red box. (c) CEACAM1 mutant cells exhibit reduced CEACAM protein expression compared to control HPMVECs by Western blot. (d) shows that successful constructions of CEACAM1^−/−^ and negative control stably transfected cell lines.

Our preliminary results showed that CEACAM1 expression is significantly increased in response to OGD/R. Therefore, we sought to establish an HPMVEC cell line lacking this gene, to assess the ability of HPMVEC cells to respond to hypoxia. Efficient lentiviral infection was followed by 30 days of puromycin selection, and high lentivirus transfection rates are shown in Figure 2(d). The stable cell lines transformed with either empty vector or the CEACAM mutant construct were assessed by PCR (Figure 2(a)), Sanger sequencing (Figure 2(b)), and Western blot (Figure 2(c)), confirming the desired mutation.

### 3.3. Detection of ROS Level and Measurement of Mitochondrial Membrane Potential by Flow Cytometry

**Figure 3:**
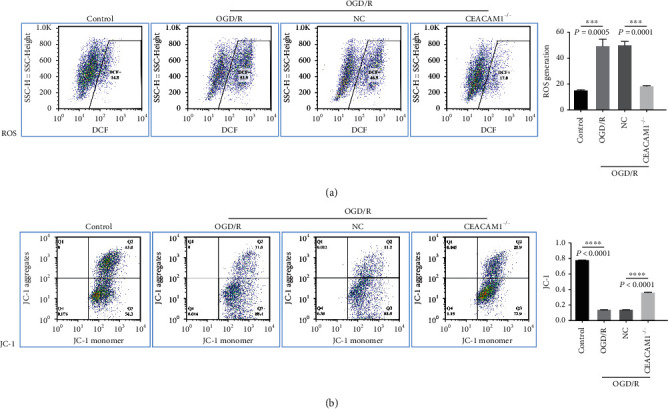
Reduction of CEACAM1 expression in HPVMECs reducing response of ROS generation and increasing mitochondrial membrane in response to hypoxic conditions. (a) Changes in cellular ROS levels in HPVMECs with and without CEACAM1 subjected to OGD/R. (b) JC-1 expression in control and CEACAM-/- HPVMECs under normal and OGD/R conditions.

Having established a cell line lacking expression of CEACAM1, we sought to test the hypothesis that CEACAM1 plays an important role in response to hypoxia. As shown in Figure 3, in the ROS experiments, the CDF^+^ of the OGD/R group (53.9) was significantly higher than that of the control group (14.8; *p* < 0.05; Figure 3(a)). In contrast, the CDF^+^ of the CEACAM1^−/−^ group (17.0) was significantly lower than either non-OGD/R control and vector only control (NC) group (46.5; *p* < 0.05). JC-1 staining revealed that the Q2 area of the control group was higher than in the OGD/R group (Figure 3(b); *p* < 0.01), and JC-1 staining was increased in CEACAM1^−/−^ cells as compared to NC (*p* < 0.01).

### 3.4. Reduced Cell Proliferation of HPMVECs under OGD/R Treatment Is Rescued by Loss of CEACAM1

**Figure 4:**
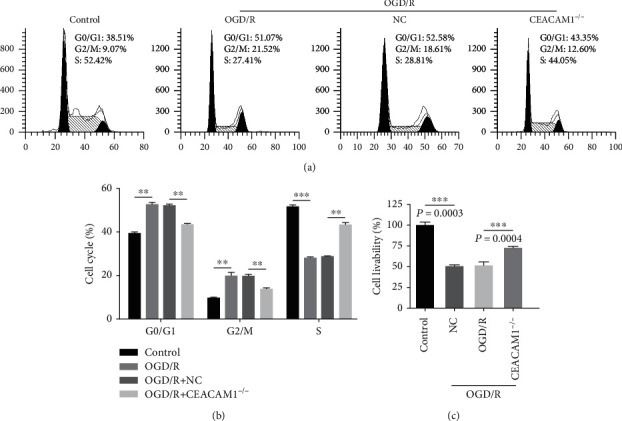
Cell cycle detection with flow cytometry and cell proliferation was measured through CCK8 assays. (a) More of the HPMVECs grown under OGD/R conditions were found to be in G0/G1 or G2/M phase, while fewer were in S phase than in the control group. (b) The cell number of CEACAM1^−/−^ group in G0/G1 or G2/M phase was reduced, while S phase increased compared to the NC group. (c) The cell viability measured by CCK8 assays of OGD/R group did not differ from the NC group, but was significantly lower than the CEACAM1^−/−^ group.

Compared to controls grown in normoxic and nonstarvation conditions, more of the HPMVECs grown under OGD/R conditions were found to be in G0/G1 or G2/M phase, while fewer were in S phase (Figure 4(a)). NC HPMVECs grown under OGD/R conditions exhibited a very similar profile to control. In contrast, CEACAM1^−/−^ HPMVECs grown under OGD/R conditions exhibited a decrease in the number of cells in G0/G1 or G2/M phase, while the number of cells in S phase was increased (Figure 4(b)). These data suggested that cell proliferation was slowed by OGD/R treatment, preventing HPMVEC cell proliferation through inhibition of S phase entry. Cell proliferation appeared to be promoted by CEACAM1 knockout, slowing down the progression of divisions. Using a CCK8 assay, we found that the cell proliferation in OGD/R group was slowed down relative to that of the control group. In contrast, we found that cell proliferation was enhanced in the CEACAM1^−/−^ group as compared to the NC controls. The above observation further supported by measurements of cell viability in each group, which showed in the model, the OGD/R group did not differ from the NC group, but was significantly lower than the CEACAM1^−/−^ group (Figure 4(c)).

### 3.5. HPMVECs Lacking CEACAM1 Exhibit Resistance to Changes in Tube Formation Competence in Response to OGD/R

**Figure 5:**
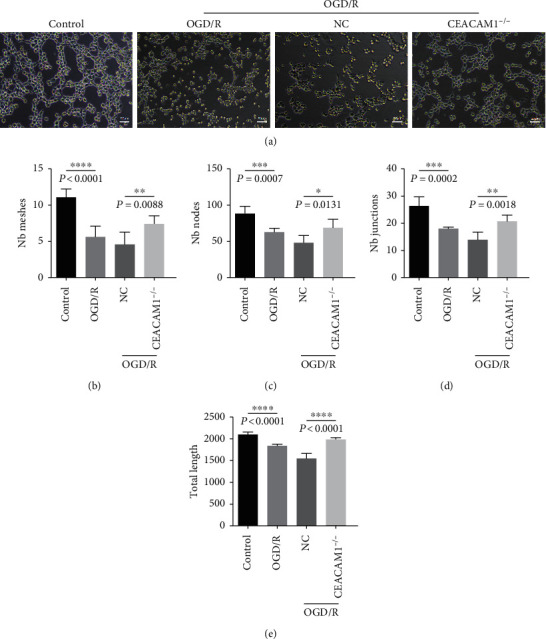
Changes in number of tubes, nodes, intersections, and total length in response to OGD/R, in control and CEACAM1^−/−^ HPVMECs. (a) Representative field of HPVMECs for each cell type, highlighting tubes measured in the tube formation assay. (b) Number of tubes formed by each cell line during the assay. (c) Number of nodes formed by each cell line during the assay. (d) Number of crossovers formed in each cell line during the assay. (e) Quantitation of tube length in each cell line during the assay.

Using tube formation as an in vitro assay of angiogenesis competency (Figure 5(a)), we found that control HPMVECs grown in normal conditions formed more tubes than the wild type HPMVECs subjected to OGD/R (Figure 5(b); *p* < 0.01), while the NC controls had considerably fewer tubes than the CEACAM1^−/−^ HPMVECs (Figure 5(b); *p* < 0.01). The number of nodes in the control group was significantly higher than that of the OGD/R group (Figure 5(c); *p* < 0.01), whereas the NC HPMVECs had far fewer nodes than the CEACAM1^−/−^ group (Figure 5(c); *p* < 0.05). Next, we counted the number of crossovers in each condition. The number of crossovers in the control group was significantly higher than observed in the OGD/R group (Figure 5(d); *p* < 0.01), while CEACAM1^−/−^ HPMVECs had more crossovers than NC controls (Figure 5(d); *p* < 0.01). The length of tubes in the control HPMVECs was longer than that of cells in the OGD/R group (Figure 5(e); *p* < 0.01), while NC HPMVEC tubes were even short; this effect was abrogated by loss of CEACAM1^−/−^ in our knockout cells (Figure 5(e); *p* < 0.01).

### 3.6. CEACAM1, TNF-*α*, VEGF, VEGFR-2, p-p38/p38, and CyclinD1 Protein Expression in OGD/R

**Figure 6:**
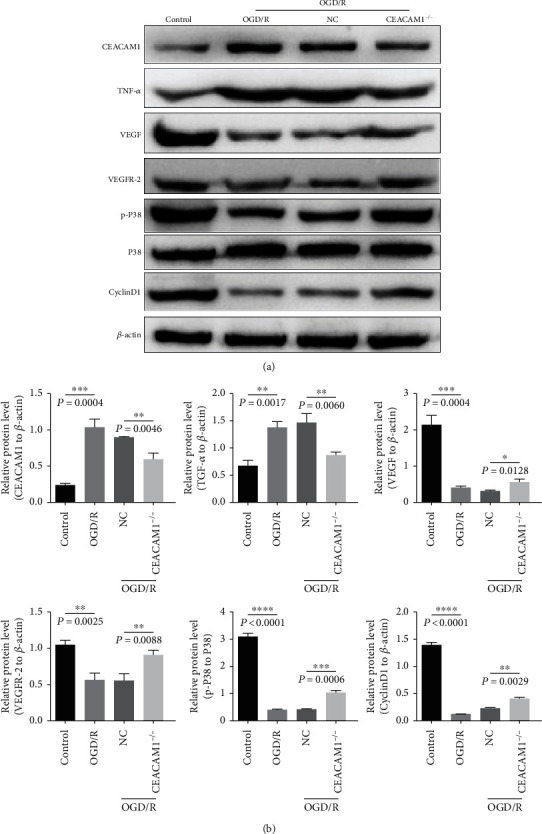
The protein levels of CEACAM1, TNF-*α*, VEGF, VEGFR-2, p-P38/P38, and CyclinD1 as detected by Western blot. (a) The expression level of CEACAM1, TNF-*α*, VEGF, VEGFR-2, cyclinD1, and MAPK phosphorylated were examined by Western blot in each group. (b) The expression level of CEACAM1 and TNF-*α* were higher while VEGF, VEGFR-2, CyclinD1, and MAPK phosphorylated were lower in the OGD/R group compared with control group; the expression level of CEACAM1 and TNF-*α* were reduced while VEGF, VEGFR-2, CyclinD1, and MAPK phosphorylated were increased in the CEACAM1^−/−^ group compared to the NC group.

The expression level of CEACAM1, TNF-*α*, VEGF, VEGFR-2, and cyclin pathway-related protein (Cyclin D1) and proliferation-associated pathway of MAPK phosphorylated were further examined by Western blot to monitor expression changes in response to our experimental treatment. Expression of CEACAM1 and TNF-*α* was higher, and levels of VEGF, VEGFR-2, Cyclin D1, and phosphorylated-MAPK were lower in OGD/R treated wild type HPVMECs than in controls (Figure 6(a), lanes 1 and 2). The opposite patterns of expression were observed in CEACAM1^−/−^ cells (Figure 6(a), lane 4) compared to NC controls (Figure 6(a), lane 3). Expressions CEACAM1 and TNF-*α* were reduced in CEACAM^−/−^ cells while VEGF, VEGFR-2, CyclinD1, and phosphorylated p38 were increased compared to the NC group (Figure 6(a); compare lanes 3 and 4). Quantitation of 3 biological replicates of this experiment is shown in Figure 6(b).

## 4. Discussion

This study reports for the first time that in a low-oxygen environment: (1) CEACAM1 expression in HPMVECs was upregulated; (2) oxidative stress was decreased by knockdown of CEACAM1; (3) knockout of CEACAM1 increased cell proliferation that is normally suppressed by low-oxygen conditions.

The pulmonary vasculature consists of three components: the arterial tree, an extensive capillary bed, and the venular tree [6]. Elevation of vascular pressure in any of these three components can lead to an increase in pulmonary vascular resistance. In human lungs, the estimated total capillary length from direct measurements of capillary profiles averages 6950 ± 3108 km [7]. The lung capillary is the primary site for alveolar gas exchange, and the loss of capillary bed is also one of the main reasons for increases in pulmonary pressure. In chronic hypoxic lung disease, the increase in pulmonary vascular resistance and subsequent pulmonary hypertension is mainly due to inflammation-induced reduction in the extent of the pulmonary vascular bed, destruction of the lung parenchyma, and vascular occlusion from the underlying disease process [8, 9]. In addition, both the structure and function of the endothelium are affected by hypoxia, and chronic hypoxic exposure causes endothelial hypertrophy and hyperplasia [10], increasing lung vascular permeability and expression of inflammatory cell markers [11]. Furthermore, hypoxia alters the surface coagulation properties of endothelial cells, inhibits normal thrombomodulin production and induces procoagulant activity [12].

In the current study, our findings suggest that knocking out CEACAM1 can reduce the oxidative stress in HPMVECs and promote cell proliferation in a hypoxic environment, which is contrary to previously published studies that the angiogenesis was promoted by CEACAM1 in tumor cells. While tumor angiogenesis is required for tumor neovascularization, which is in turn essential for tumor spread and growth, the function of CEACAM1 in tumors remains unclear. CEACAM1 expression has been reported to be lower in various carcinomas, such as urinary bladder cancer, renal cell carcinomas, and prostate cancer, as compared to nontumor tissue [13–15]. Paradoxically, CEACAM1 expression has been reported to be increased in several other cancers, including gastric cancer and nonsmall-cell lung cancer [16–18]. One consistent finding about CEACAM1 is that it contributes to vascular stabilization and maturation by regulating both endothelial tube formation and the integration of smooth muscle cells into the wall of newly formed vessels [19–21]. Ergun et al. found that using pure, soluble CEACAM1 from human granulocytes, CEACAM1 promoted angiogenesis by improving endothelial cell migration and tube formation, as well as by increasing vessel density in chorion allantois membrane experiments in vivo [19].

In the context of myocardial infarction (MI), Wang et al. recently found that CEACAM1 expression was elevated in cardiomyocytes after MI, suggesting a potential role in repair. Moreover, in the same study, CEACAM1^−/−^ mice were found to have improved cardiac function, less cardiac remodeling and fibrosis, and reduced cardiomyocyte apoptosis after MI compared to wild type mice, resulting in lower mortality [22]. That report is consistent with our results from the current study, which showed that knockdown of CEACAM1 has a protective effect on HPMVECs under hypoxic conditions. We also observed that by knocking out CEACAM1, HPMVECs more easily entered S phase in a hypoxic environment, promoting cell proliferation, and promoted tube formation in our in vitro model of angiogenesis. This may be related to downregulation of TNF-*α* and upregulation of VEGF, VEGFR-2, CyclinD1, and MAPK phosphorylation level. It suggests that the roles of CEACAM1 in tumor and normal tissues may be different. Interestingly, previous studies have suggested a link between VEGF-induced angiogenesis and CEACAM1 function. In vitro, siRNA-mediated CEACAM1 knockdown in endothelial cells reduces VEGF-induced angiogenesis, and data from CEACAM1^−/−^ mice suggest that CEACAM1 is a downstream effector of VEGF [23].

## 5. Limitations of the Current Study

There were several important limitations of the current study. First, our study has only reported that oxidative stress was decreased, and cell proliferation was enhanced by knockdown of CEACAM1 in HPMVECs under hypoxia using cultured cells. It is still unclear whether CEACAM1 functions in the same way in these processes in hypoxic conditions at the organismal level. Second, CEACAM1 is a transmembrane molecule that has three domains: an extracellular domain, a transmembrane domain, and a cytoplasmic domain. The latter is a signaling molecule contains immunoreceptor tyrosine-based inhibitory patterns. At the cellular level, more research is needed to determine how CEACAM1 mediates the oxidative stress and proliferation suppression of pulmonary capillary endothelial cells generated by hypoxia.

## Data Availability

The data used to support the findings of this study are included within the article.
